# Preemptive targeted muscle reinnervation: the single incision approach should be avoided in trans-tibial traumatic amputation

**DOI:** 10.1186/s40779-022-00421-z

**Published:** 2022-10-24

**Authors:** Laurent Mathieu, Constance Diner, Philippe Aries, Marie Thomas, Stéphanie Truffaut, Nicolas de L’escalopier

**Affiliations:** 1 Department of Orthopedic, Trauma and Reconstructive Surgery, Percy Military Hospital, 101 avenue Henri Barbusse, 92140 Clamart, France; 2 French Military Hand Surgery Center, Percy Military Hospital, 92140 Clamart, France; 3 Department of Surgery, Ecole du Val-de-Grâce, French Military Health Service Academy, 75005 Paris, France; 4 Department of Anesthesia and Intensive Care, Clermont-Tonnerre Military Hospital, 29240 Brest, France; 5 Department of Rehabilitation Medicine, Percy Military Hospital, 92140 Clamart, France; 6 Department of Rehabilitation Medicine, National Institution of Invalids, 75007 Paris, France

**Keywords:** Heterotopic ossification, Nerve transfer, Targeted muscle reinnervation, Trans-tibial amputation, Trauma

Dear Editor,

Chronic pain is a significant concern after major lower limb amputations that often preclude prosthetic fitting, decrease ambulation, and impact the quality of life [1, 2]. In the last decade, targeted muscle reinnervation (TMR) has been proposed as a surgical strategy for treating or preventing symptomatic neuromas and phantom-limb phenomena in major amputees [1]. This technique involves the transfer of an amputated mixed-motor and sensory nerve to a nearby recipient motor nerve [1, 2]. Unlike most surgical strategies that aim to hide or protect the neuroma, TMR gives the amputated nerves “somewhere to go and something to do” [2]. In a randomized clinical trial on neuroma and phantom pain, Dumanian et al. [1] demonstrated that TMR reduces amputation-related chronic pain at 1-year post-intervention when compared with the excision and muscle-burying technique, which remains the current gold standard. Valerio et al. [2] also proposed applying TMR at the time of major limb amputation for preventing chronic pain and found that TMR patients experienced less residual limb pain (RLP) and phantom limb pain (PLP) when compared with untreated amputee controls.

In our practice, TMR is routinely used to treat neuroma and PLP with the same favorable results as those reported in the current literature. With this experience and based on the outcomes published by Valerio et al. [2], we have applied TMR as a preemptive procedure to limit the occurrence of chronic pain following lower limb traumatic amputations. An institutional study was then conducted to confirm that preemptive TMR is a safe and beneficial strategy as compared to traditional amputation conducted with traction neurectomy alone (Additional file 1).

Between 2019 and 2020, 10 patients received TMR at the time of lower limb traumatic amputations including 6 trans-tibial amputees with a median age of 37.0 years (Additional file 2: Table S1). Among the latter, TMR was applied to the mixed amputated nerves (tibial nerve, deep and superficial fibular nerves) using a single incision through the wound as proposed by Bowen et al. [3] (Additional file 3: Fig. S1). Since delayed primary closure was required in 4 patients, the median time from amputation to TMR was 6 d. Late complications requiring reoperation occurred in 4/6 trans-tibial amputees and were related to TMR in 3/6 patients. There were deep or superficial fibular nerve transfer entrapments by heterotopic ossification or scar tissue that precluded prosthetic fitting (Fig. 1). All required elective revision neurectomy together with ossification excision when necessary. Such complications have naturally altered TMR outcomes, but all patients made it to prosthesis at the last follow-up (Additional file 2: Tables S1 and S2; Additional file 3: Figs. S2 and S3). Considering these frequent failures, we modified the surgical protocol to avoid nerve transfer completion adjacent to the fibular osteotomy.

**Fig. 1:**
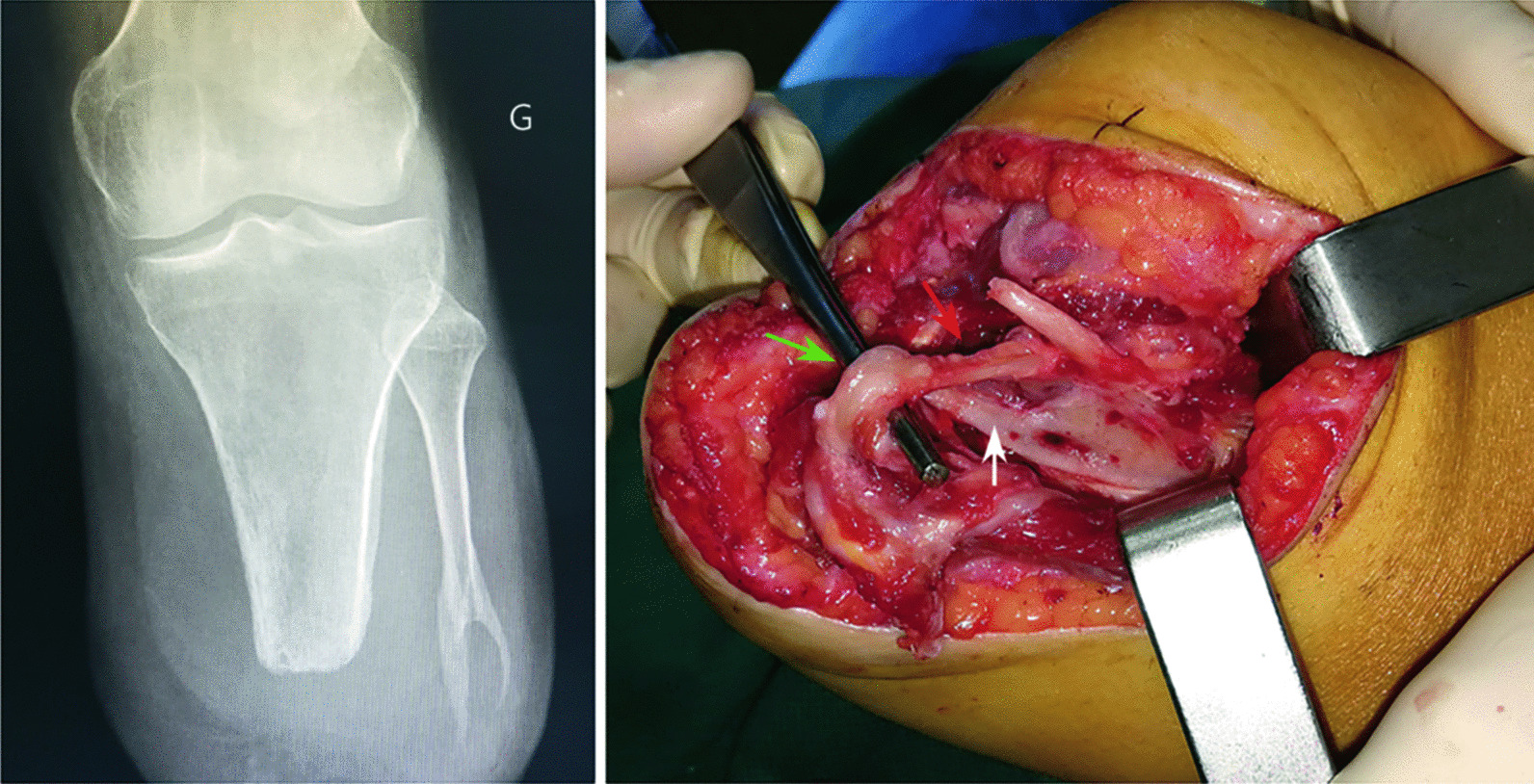
Nerve transfer entrapment due to heterotopic ossification from the distal fibula: radiological aspect (right) and intraoperative view (left). The suture site between the donor nerve (superficial fibular nerve—green arrow) and the recipient nerve (long fibular muscle motor branch—red arrow) was found entrapped inside the heterotopic ossification (white arrow)

Our preliminary results contrast with those of the existing literature, although clinical studies reporting on preemptive TMR are few [2, 4]. In the present study, two patients developed a similar ossification at the end of the fibula, with the entrapment of a nerve transfer inside the newly formed bone (Fig. 1). Another one had no ossification but suffered from a severe irritative syndrome on the two nerve transfers performed close to the fibula osteotomy. Following revision neurectomy (performed after the 1-year assessment) these patients experienced substantial or complete pain relief and had successful prosthetic fitting within a few weeks. Only Anderson et al. [5] recently reported a similar case of TMR disruption due to heterotopic ossification in a trans-tibial amputee. We believe that the single-incision technique in the setting of trans-tibial traumatic amputation might favor distal nerve transfer entrapment in scar tissue or heterotopic ossification [3]. Such complications could also be favored by delayed stump closure. In any cases, it seems important to avoid nerve coaptation near or distal to the fibula osteotomy as proposed by Chang et al. [4]. A two-incision approach is likely to prevent such complications by avoiding extensive soft tissue dissection and nerve transfer performed in an injured area [4]. It is in fact easier and safer to perform TMR from a second proximal incision where motor nerves can be clearly identified, rather than from inside the wound where inflamed tissue planes must be opened looking for motor nerves either branching off the major mixed nerve or found within muscle.

To conclude, we aimed to stress that TMR performed at the time of trans-tibial traumatic amputation should be used with caution. Nerve transfer entrapment within the injured area may jeopardize TMR effects. Such a complication seems to be related to nerve transfers performed adjacent to the fibular osteotomy site. Thus, we believe that performing trans-tibial amputation and TMR through a single incision should be avoided. A technique using a second incision behind the knee could avoid such pitfalls and facilitate nerve transfer completion.

## Supplementary Information

**Additional file 1**. Materials and Methods.

**Additional file 2. Table S1**. Comparison of the baseline characteristics, follow-up time and postoperative complications between two groups.** Table S2**. Comparison of the NRS and PROMIS scores for RLP and PLP at the last follow-up between two groups [median (IQR)].

**Additional file 3. Fig. S1**. Surgical protocol for TMR in trans-tibial traumatic amputation.** Fig. S2**. NRS scores evolution in the first year following TMR.** Fig. S3**. PROMIS scores evolution in the first year following TMR.

## Data Availability

Data are available on reasonable request.

## Abbreviations

PLP: Phantom limb pain
RLP: Residual limb pain
TMR: Targeted muscle reinnervation
