# Supplementary material for: Preemptive targeted muscle reinnervation: the single incision approach should be avoided in trans-tibial traumatic amputation
Source: Mil Med Res. 2022 Oct 24;9:60. doi: 10.1186/s40779-022-00421-z (PMC9590119; doi:10.1186/s40779-022-00421-z)
Supplement: Supplementary file 1 — Additional file 1. Materials and Methods. [file 40779_2022_421_MOESM1_ESM.pdf]

Materials and Methods

The purpose of our comparative study was to evaluate targeted muscle reinnervation (TMR) as a preemptive treatment of residual limb pain (RLP) and phantom limb pain (PLP) at the time of major lower-limb amputations for traumatic reasons. We hypothesized that this strategy would be safe and more beneficial as compared to traditional amputation conducted with only neurectomy.

Population studied

TMR group

After obtaining approval from our institutional review board (Protocol Number: 42-2019 HIA-CS), a prospective study was conducted on patients who underwent traumatic trans-tibial or trans-femoral amputation between 2019 and 2020. All of them were offered a consultation to undergo concurrent TMR and agreed to the procedure. This consultation occurred prior to the scheduled amputations for those with chronic or subacute injuries and prior to stump closure for those with acute injuries treated by emergent amputation with delayed primary closure. These patients were managed by the first senior author. The inclusion criteria comprised patients over 17 years of age who underwent TMR at the time of primary or revision amputation. The exclusion criteria comprised patients who scheduled for non-traumatic amputation, or cognitively impaired. The minimum follow-up time for this group was 12 months.

Conventional amputation group

The sample of conventional amputees without TMR was identified among the patients managed at our rehabilitation center for prosthetic fitting in 2021. We did not capture prospectively these patients to maximize enrollment in the TMR group. These patients were operated by various senior surgeons. The data were collected through a survey proposed during outpatient treatment. The inclusion criteria comprised patients over 17 years of age who underwent traumatic trans-

tibial or trans-femoral amputation without TMR and successfully completed the survey. The exclusion criteria comprised patients who were amputated for non-traumatic indications, or submitted incomplete or duplicate survey responses. The minimum follow-up time for this group was 3 months.

Surgical technique

Patients of both groups underwent the same surgical protocol regarding skin incision, fat flaps elevation, muscles debulking and stump coverage using myodesis or myoplasty. Only amputated nerves management varied between groups. In the postoperative period, all patients were managed by the same rehabilitation team for prosthetic fitting.

TMR group

TMR was applied to the mixed amputated nerves within the wound in trans-tibial amputees as described by Bowen et al. [1] (Additional file 3: Fig. S1) or through a second proximal incision in trans-femoral amputees as proposed by Agnew et al. [2].

Conventional amputation group

In this group, amputated nerves were addressed using traction neurectomy and muscle implantation following an intraneural injection of ropivacaine.

Data analyzed

Perioperative data

For both groups, we collected data regarding demographics, injury mechanism, amputation level (trans-tibial or trans-femoral), amputation type (primary or revision), and complications requiring reoperation.

Chronic pain assessment

First, TMR patients and conventional amputees were asked to rate their worst, best, and current pain level over the past 24 h on a 0–10 Numerical Rating Scale (NRS) [3]. Next, RLP and PLP were assessed using four Patient-Reported Outcome Measurement Information System (PROMIS) instruments: PROMIS pain intensity – Short Form 3a, Neuropathic pain quality – Short Form 5a, pain behavior – Short Form 7a, and pain interference – Short Form 8a [4-7].

Statistical analysis

The data were collected using Microsoft Excel (Microsoft Corp., Redmond, WA, USA). The categorical variables were compared using the chi-square test or Fisher's exact test. After testing for normality (Kolmogorov-Smirnov test) and for equality of variances (Fisher-Snedecor test), the continuous variables were compared either by Student's *t*-test or Wilcoxon's rank-sum test. For assessing chronic pain, we used the Friedman test, which is a non-parametric test for repeated measures. The tests were two-sided, and values of P less than 0.05 were considered statistically significant. The statistical analysis was implemented using R (1.1.463).

References

1. Bowen JB, Ruter D, Wee C, West J, Valerio IL. Targeted muscle reinnervation technique in below-knee amputation. Plast Reconstr Surg. 2019;143(1):309-12.

2. Agnew SP, Schultz AE, Dumanian GA, Kuiken TA. Targeted reinnervation in the transfemoral amputee: A preliminary study of surgical technique. Plast Reconstr Surg. 2012;129(1):187-94.

3. Sallaffi F, Stancati A, Silvestri CA, Ciapetti A, Grassi W. Minimal clinically important changes in chronic musculoskeletal pain intensity measured on a numerical rating scale. Eur J Pain. 2004;8(4):283-91.

4. Chen WH, Revicki D, Amtmann D, Jensen MP, Keefe FJ, Cella D. Development and analysis of PROMIS pain intensity scale. Qual Life Res. 2012;20(Suppl 1):18.

5. Askew RL, Cook KF, Keefe FJ, Nowinski CJ, Cella D, Revicki DA, et al. A PROMIS measure of neuropathic pain quality. Value Health. 2016;19(5):623-30.

6. Revicki DA, Chen WH, Harnam N, Cook KF, Amtmann D, Callahan LF, et al. Development and psychometric analysis of the PROMIS pain behavior item bank. Pain. 2009;146(1-2):158-69.

7. Amtmann D, Cook KF, Jensen MP, Chen WH, Choi S, Revicki D, et al. Development of a PROMIS item bank to measure pain interference. Pain. 2010;150(1):173-82.
