# Supplementary material for: Preemptive targeted muscle reinnervation: the single incision approach should be avoided in trans-tibial traumatic amputation
Source: Mil Med Res. 2022 Oct 24;9:60. doi: 10.1186/s40779-022-00421-z (PMC9590119; doi:10.1186/s40779-022-00421-z)
Supplement: Supplementary file 2 — Additional file 2. Table S1. Comparison of the baseline characteristics, follow-up time and postoperative complications between two groups. Table S2. Comparison of the NRS and PROMIS scores for RLP and PLP at the last follow-up between two groups [median (IQR)]. [file 40779_2022_421_MOESM2_ESM.pdf]

Table S1 Comparison of the baseline characteristics, follow-up time and postoperative complications between two groups

**Variable**	**TMR group** (***n*** **= 10 patients, 10 limbs)**	**Conventional amputation group** (***n*** **= 18 patients, 20 limbs)**	***P*-value**
Age [years, median (IQR)]	37.0 (29.0 – 51.0)	30.0 (27.0 – 36.5)	0.12
Male [*n* (%)]	8 (80.0)	18 (100.0)	
Injury mechanism [*n* (%)]			0.88
Crash	7 (70.0)	14 (77.8)	
Ballistic trauma	2 (20.0)	2 (11.0)	
Work accident	0	1 (5.6)	
Sports accident	1 (10.0)	1 (5.6)	
Amputation level [*n* (%)]			0.58
Trans-tibial	6 (60.0)	16 (80.0)	
Trans-femoral	4 (40.0)	4 (20.0)	
Amputation type [*n* (%)]			0.73

Variable	TMR group<br>(*n* = 10 patients, 10 limbs)	Conventional amputation group<br>(*n* = 18 patients, 20 limbs)	*P*-value
Primary	7 (70.0)	17 (85.0)	
Revision	3 (30.0)	3 (15.0)	
Complications requiring revision [*n* (%)]			0.70
Heterotopic ossification	2 (20.0)	2 (10.0)	
Unstable soft tissue coverage	0	4 (20.0)	
Painful neuroma	2 (20.0)	2 (10.0)	
Other	1 (10.0)	0	
Follow-up time [months, median (IQR)]	14.5 (13.0 – 21.0)	26.0 (10.2 – 46.5)	0.55

Ten TMR patients included prospectively were compared to 18 conventional amputees – included retrospectively during the same period. The categorical variables were compared using the chi-square test or Fisher's exact test. After testing for normality and equality of variances, the continuous variables were compared either by Student's *t*-test or Wilcoxon's rank-sum test. *TMR* targeted muscle reinnervation

Table S2 Comparison of the NRS and PROMIS scores for RLP and PLP at the last follow-up between two groups [median (IQR)]

**Variable**	**TMR group** (***n*** **= 10 patients, 10 limbs)**	**Conventional amputation group** (***n*** **= 18 patients, 20 limbs)**	***P*-value**
NRS scores			
Worst RLP	1.5 (0 – 4.7)	3.0 (2.0 – 4.0)	0.61
Best RLP	0 (0 – 0)	0 (0 – 2)	0.31
Current RLP	1 (0 – 2)	0 (0 – 3)	0.96
Worst PLP	1.5 (0.2 – 5.0)	4.0 (0 – 5.7)	0.86
Best PLP	0 (0 – 0.7)	0 (0 – 0.7)	0.83
Current PLP	0.5 (0 – 2.7)	0 (0 – 2.0)	0.64
PROMIS scores			
RLP intensity	36.3 (32.0 – 46.0)	46.3 (41.0 – 52.0)	0.18
RLP quality	49.6 (43.0 – 53.0)	51.8 (46.0 – 56.0)	0.60
RLP behavior	53.4 (43.0 – 55.0)	57.3 (49.0 – 61.0)	0.31

Variable	TMR group (*n* = 10 patients, 10 limbs)	Conventional amputation group (*n* = 18 patients, 20 limbs)	*P*-value
RLP interference	52.7 (48.0 – 57.0)	51.7 (41.0 – 61.0)	0.85
PLP intensity	41.8 (33.1 – 48.0)	47.8 (31.0 – 52.0)	0.64
PLP quality	49.6 (39.5 – 55.0)	49.6 (37.0 – 56.0)	0.98
PLP behavior	51.1 (36.0 – 54.0)	52.8 (34.0 – 57.0)	0.66
PLP interference	49.9 (42.0 – 55.0)	40.7 (41.0 – 57.0)	0.61

After testing for normality and equality of variances, these variables were compared either by Student's *t*-test or Wilcoxon's rank-sum test. *NRS* numerical rating scale, *PLP* phantom limb pain, *PROMIS* patient-reported outcomes measurement information system, *RLP* residual limb pain, *TMR* targeted muscle reinnervation
