# Supplementary material for: Preemptive targeted muscle reinnervation: the single incision approach should be avoided in trans-tibial traumatic amputation
Source: Mil Med Res. 2022 Oct 24;9:60. doi: 10.1186/s40779-022-00421-z (PMC9590119; doi:10.1186/s40779-022-00421-z)
Supplement: Supplementary file 3 — Additional file 3. Fig. S1. Surgical protocol for TMR in trans-tibial traumatic amputation. Fig. S2. NRS scores evolution in the first year following TMR. Fig. S3. PROMIS scores evolution in the first year following TMR. [file 40779_2022_421_MOESM3_ESM.pdf]

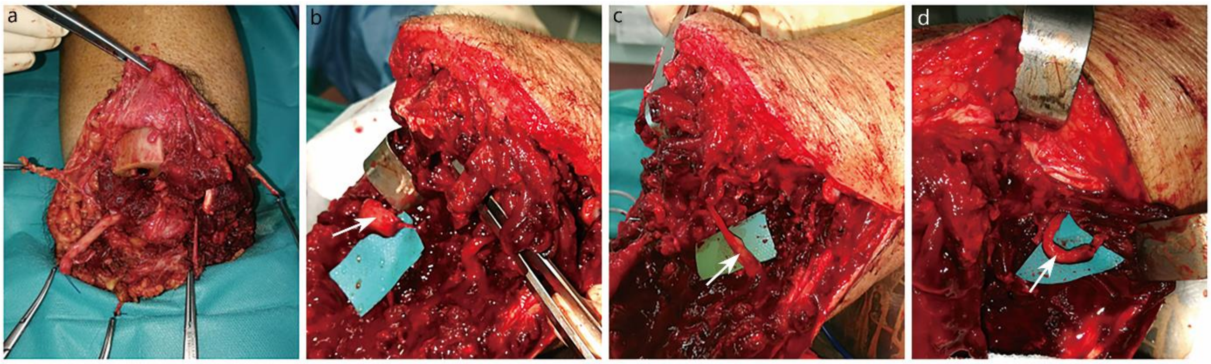


**Fig. S1** Surgical protocol for TMR in trans-tibial traumatic amputation. **a** The mixed amputated nerves were dissected through the wound in a single incision approach, then excised to healthy nerve fascicules. The motor nerves innervating the nearby muscles that had been rendered functionless by the amputation were identified using a nerve stimulator; these nerves were intended to serve as potential recipients. The amputated nerve (donor's nerve) was then sutured in an end-to-end manner to the surgically divided distal segment of the motor nerve (recipient's nerve). **b**-**d** In this case, the tibial nerve (white arrow) was transferred to a motor branch of the tibialis posterior muscle (**b**), the deep fibular nerve (white arrow) was transferred to a motor branch of the tibialis anterior muscle (**c**), the superficial fibular nerve (white arrow) was transferred to a motor branch of the fibular muscles (**d**). Conversely, TMR was not applied to pure sensory nerves. The sural nerve was addressed by an end-to-side suture to the nearby mixed donor nerve, and the saphenous nerve was treated using traction neurectomy

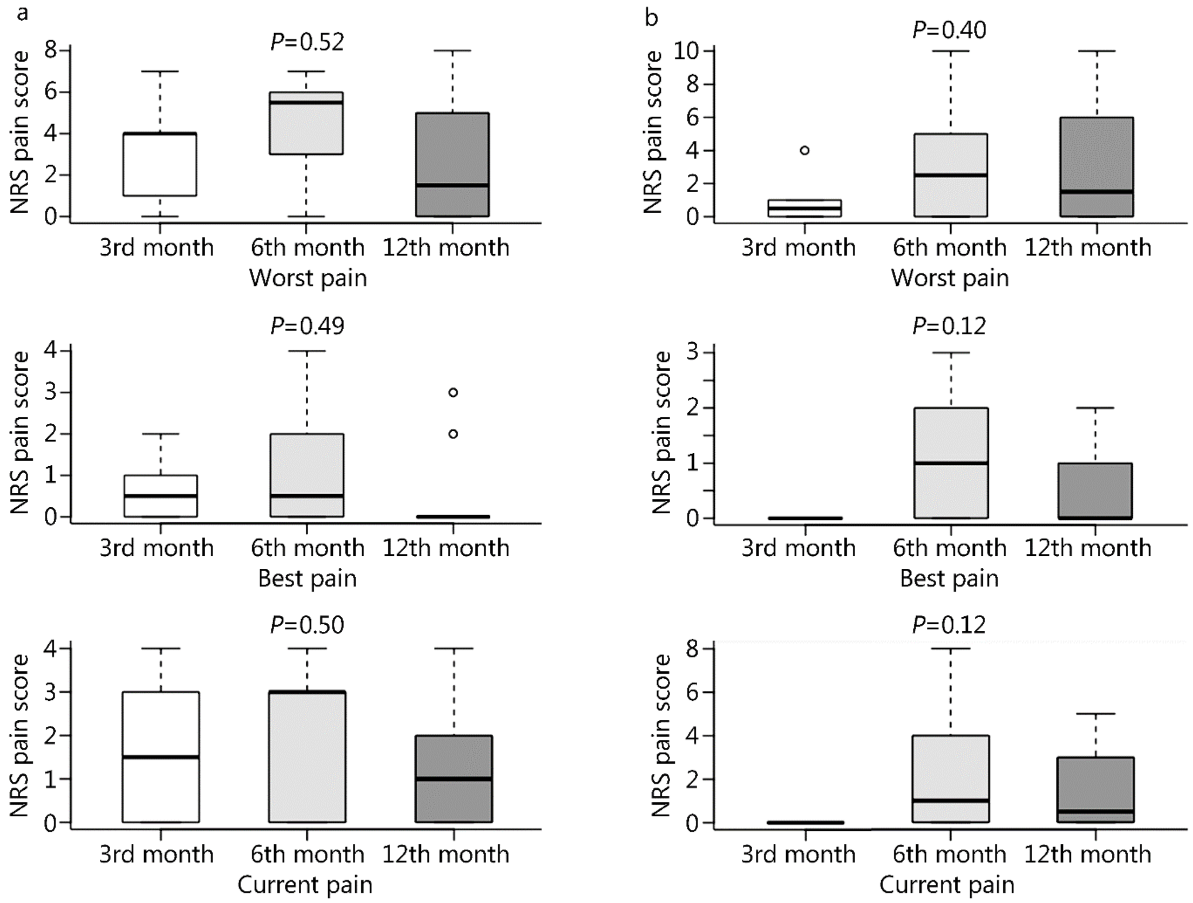


**Fig. S2** NRS scores evolution in the first year following TMR. **a** NRS scores of residual limb pain (RLP). **b** NRS scores of phantom limb pain (PLP). A non-parametric Friedman test was used for repeated measures. Values of *P* less than 0.05 were considered statistically significant. No significant differences were recorded over time for either RLP or PLP. NRS numerical rating scale, TMR targeted muscle reinnervation

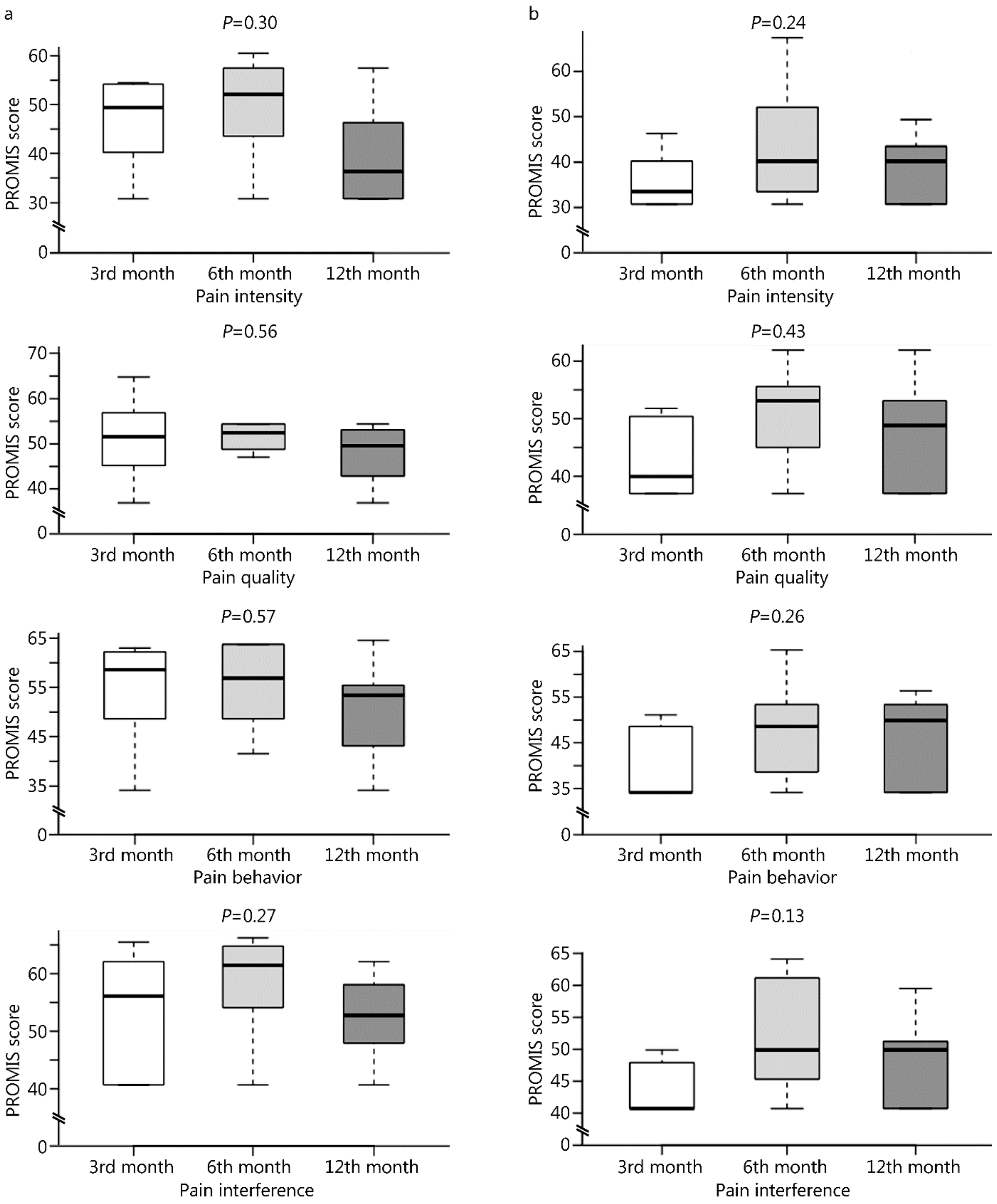


**Fig. S3** PROMIS scores evolution in the first year following TMR. **a** NRS scores of residual limb pain (RLP). **b** NRS scores of phantom limb pain (PLP). A non-parametric Friedman test was used for repeated measures. Values of *P* less than 0.05 were considered statistically significant. No significant differences were recorded over time for either RLP or PLP. PROMIS patient-reported outcomes measurement information system, TMR targeted muscle reinnervation
